# CONFLICTS BETWEEN OLDER ADULTS AND THEIR CAREGIVERS DURING ACUTE HOSPITALIZATION: AN ECOLOGICAL PERSPECTIVE

**DOI:** 10.1093/geroni/igae098.2726

**Published:** 2024-12-31

**Authors:** Tova Band-Winterstein, Malek Masarwa, Anna Zisberg, Efrat Shadmi, Nurit Gur-Yaish, Ksenya Shulyaev

**Affiliations:** University of Haifa, Haifa, HaZafon, Israel; Edith Wolfson Medical Center, Jerusalem, Yerushalayim, Israel; University of Haifa, Haifa, Hefa, Israel; University of Haifa, Haifa, Hefa, Israel; Oranim Academic College, Kiryat Tivon, HaZafon, Israel; University of Haifa, Haifa, Hefa, Israel

## Abstract

Family support is important for older adults during hospitalization; however this stressful period can exacerbate or precipitate conflicts between patients and their caregivers. We aimed to identify predictors of conflicts between hospitalized older adults and family caregivers across individual, social, and cultural levels of ecological model, utilizing data from prospective HOPE-MORe Study. The sample comprised 583 cognitively intact older adults (65+) admitted to internal units in Israeli hospitals. Additionally, questionnaires filled 73 family caregivers of these patients. 29.3% of older adults and 27.4% caregivers reported some kind of conflict during hospitalization. Multiple logistic regression analysis revealed several significant predictors of conflicts. Female patients’ gender emerged as a protective factor (β=.607, 95% CI [.409,.901]), while an increased number of visitors (β=1.044, 95% CI [1.001,1.089]) and family involvement in instrumental care (β=1.221, 95% CI [1.018,1.466]) were associated with a higher likelihood of conflict. Additionally, being an immigrant from the ex-USSR was linked to an increased risk of conflict (β=1.692, 95% CI [1.069,2.679]). Notably, other factors such as age, functional and cognitive status, anxiety, duration of visits, or Arab ethnicity were found to be non-significant in predicting conflicts. Among subsample of caregivers, spousal caregivers (β=7.390 (95%CI [1.593,34.287])) and those with anxiety attachment (β=1.628 (95%CI [1.029,2.578])) were more likely to report conflict. Findings highlight the importance of ecological perspective considering individual, social, and cultural conflict risk factors. Moreover, it is important to consider both family caregivers’ and patients’ perspectives of conflict as risk factors exist on both sides, leading to a more holistic picture.

